# Evaluation of neo-implant abutment junction sealing gel among Indian patients

**DOI:** 10.6026/97320630019502

**Published:** 2023-04-30

**Authors:** Saumya Mehta, Sahana Selvaganesh, Thiyaneswaran Nesappan, Vishnu Priya Veeraraghavan, Palanivel Sathishkumar, Rajalakshmanan Eswaramoorthy

**Affiliations:** 1 Department of Implantology, Saveetha Dental College and Hospitals, Saveetha Institute of Medical and Technical Sciences (SIMATS), Saveetha University, Chennai 600077, India; 2 Department of Biochemistry, Saveetha Dental College and Hospitals, Saveetha Institute of Medical and Technical Sciences (SIMATS), Saveetha University, Chennai 600077, India; 3 Department of Biomaterials (Green lab), Saveetha Dental College and Hospital, Saveetha Institute of Medical and Technical Sciences (SIMATS), Saveetha University, Chennai 600077, India

**Keywords:** Implant abutment junction, micro-leakage, sealing gel

## Abstract

With the advent of various implant abutment junctions, the ultimate aim is to develop a precise implant abutment junction with negligible micro-leakage. However precise the mechanical connection is, there seems to be a negligible amount of micro-leakage that is present that can be addressed with the help of sealing gel. This study aims to assess the micro-leakage between the neo-sealing gel and the commercially available implant sealing gel. The study was conducted on implants (n=15) with internal hex connections, group 1 (No gel, n=5), group 2 (Neo gel, n=5) and group 3 (commercial gel, n=5). Mean dE*ab values of 0.28± 0.02, 0.04± 0.01 and 0.17±0.01 were noted for the 3 groups, and there was statistically significant difference between the 3 groups (p≤0.05). The study suggests that the neo sealing gel may be a promising material to prevent bacterial ingress and micro-leakage at the implant abutment junction.

## Background:

The small gaps or defects at the IAJ allow for bacterial penetration and accumulation within the peri-implant tissues, leading to inflammation and bone loss [1] reported that the presence of micro-leakage at the IAJ significantly increased the risk of developing peri-implantitis, Similarly, another study [2] found that the degree of bacterial contamination in the peri-implant sulcus was significantly higher in implants with micro gaps at the IAJ compared to those without micro gaps. A systematic review reported that the use of a gel at the IAJ significantly reduced the incidence of peri-implantitis compared to control groups [3]. In another systematic review and meta-analysis [4,5] platform switching was found to significantly reduce IAJ micro-leakage . Other studies have reported mixed results, with some showing no significant difference compared to conventional implant-abutment connections [6, 7]. Additionally, studies have suggested that MAJI may improve the seal at the IAJ and reduce micro-leakage [8]. The implant-abutment connection type significantly affected crestal bone loss and implant failure, with internal conical connections associated with lower bone loss compared to external hex connections [9]. It was found that platform switching reduced marginal bone loss and provided more prosthetic space. Platform switching and MAJI show promise for reducing IAJ micro-leakage and improving implant outcomes [10]. Therefore, it is of interest to document the evaluation of neo implant abutment junction sealing gel.

## Materials and Methods:

**Figure 1:**
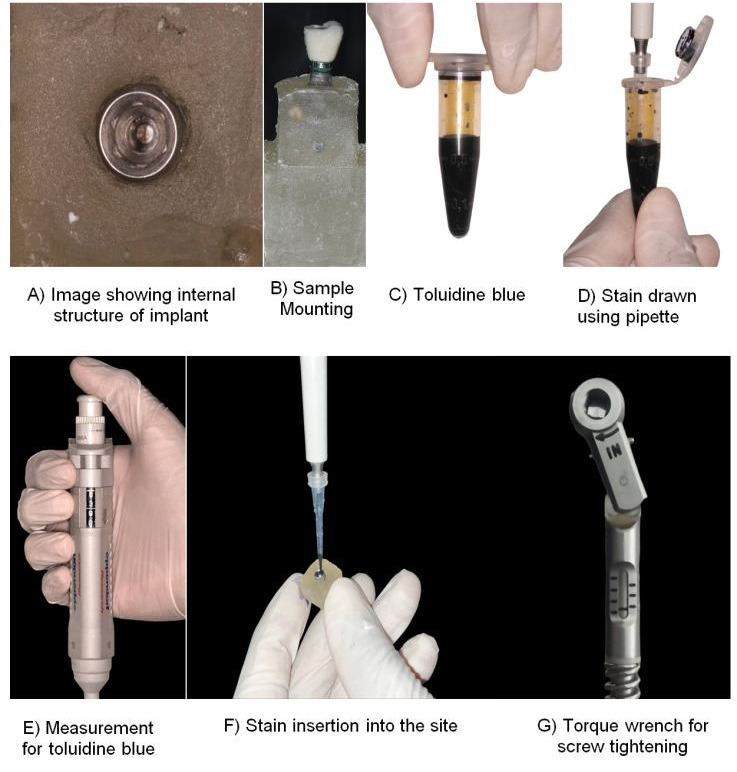
Sample preparation for testing under Dynamic loading.

An In-Vitro study was designed to be conducted at the Department of Implantology, Saveetha Dental College and Hospitals. The study was approved by the Institutional Review Board of Saveetha Dental College, Chennai, India. This study was designed as described elsewhere [11, 12, 13, 14, 15, 16, 17, 18, 19, 20]. Sterilisation of implants and abutments was accomplished by using Gamma Irradiation and all the supplementary materials and instruments were autoclaved at 121 C for 20 minutes and sterility was ensured by pre-tests .All the tests which were done were performed in bio safety cabinet for avoiding cross contamination. A total of 15 samples were prepared (5 in each of the 3 group) for this dynamic loading test which included mounting of implant into an auto polymerizing acrylic resin such that crestal 2-3 mm of implant platform lies outside of the mount. After mounting implant to specific required level, abutments were screwed to the implant to check the level of implant abutment junction. Screw retained prosthesis was fabricated for each sample which could easily be screwed to the implant. Using a pipette with a small diameter tip, 3 mL of toluidine blue (TB) solution was injected into the deepest area of the interior compartment of implant system before the components were assembled. Neo sealing gel was formulated by the premix Poly-dimethyl siloxane (PDMS) solution was prepared using sylgard 80 The antibiotic gel formulation was prepared by mixing PDMS (1%) gel with 1 mg/mL concentration of metronidazole. The resulting antibiotic-PDMS gel solution was stored at 4°C until use. Neo Sealing gel and commercially available gel was applied on the top portion of the platform before torquing the crown cemented abutment to the implant. After gel application abutment was torqued to 20Nm using a torque wrench and gel was applied at the implant abutment junction [Figure 1].

## Dynamic loading test:

**Figure 2:**
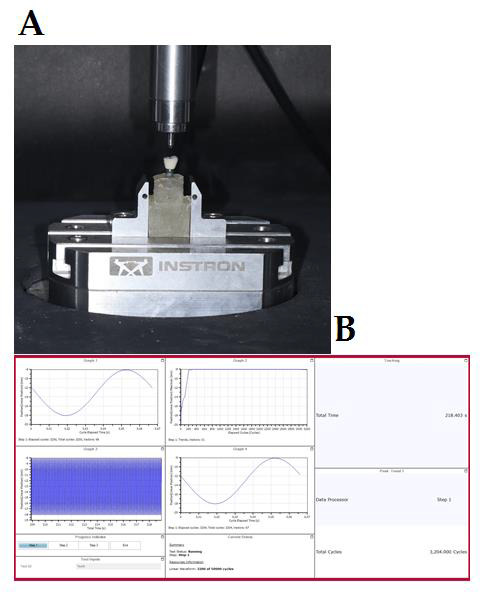
sample mounted on platform for dynamic testing. a) Instron E3000 UTM dynamic testing. b) Wave matrix analysing software

The specimens were set into a holder with the implant's long axis and indenter at a 90-degree angle. The IAI connection was submerged in 80 mL of distilled water to replicate the oral environment. Using a mechanical testing machine (E-3000 UTM), a dynamic load of 200 N with a frequency of 15 Hz was applied to the specimens while they were under load control (figure 2A). The experiment, which replicated 50,000 mastication force cycles, was run for 1 hour on each specimen at room temperature of 25 C (figure 2A).Cycles were observed using Instron wave matrix software (figure 2B). After the completion of the dynamic loading, samples were immersed in 80 ml of distilled water for 48 hours.

## Spectrophotometry testing:

**Figure 3:**
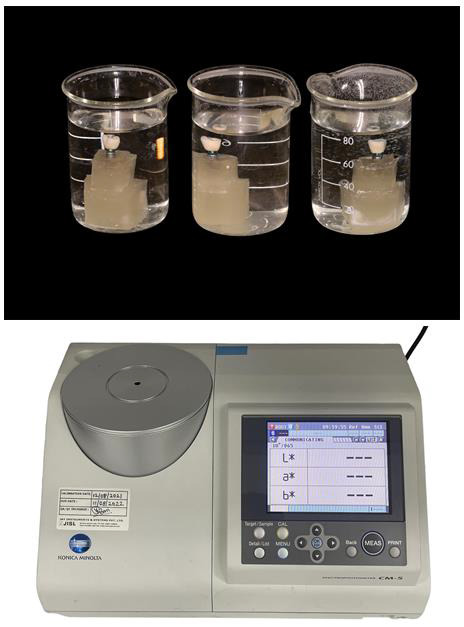
Sample immersed in distilled water and colour analysis done using spectrophotometer (Konica Minolta CM5)

After 48 hours, the Toluidine solution that was released into the distilled water media was quantitatively examined using a spectrophotometer (Konica Minolta CM5 Spectrophotometer) (Figure 3) and its values were tabulated.

## Statistical analysis:

The data was analysed to be non-parametric after subjecting to a test of normality. The comparison between Control (Group 1), Commercial gel (Group 2) and Neo sealing gel (Group 3) was done using one way ANOVA. For multiple comparisons Tukey Post Hoc test was performed. The mean percentage was tabulated. The percentage was represented graphically. The statistical analysis was calculated using SPSS VERSION 20.0 to test significance at a 5% level.

## Results:

## Dynamic loading and spectrophotometry:

A total of 3 groups with 5 samples each were evaluated, which showed mean dE*ab values of 0.28± 0.02, 0.04± 0.01 and 0.17±0.01 for group 1 (No gel), group 2 (Neo gel) and group 3 (commercial gel) respectively which was calculated using L*,a*,b* values (Table 1).Each of the three values that the CIE (International Commission on Illumination) LAB colour space use to measure objective colour and determine colour differences is denoted by the letters L*, a*, and b*. A* and b* represent chromaticity with no defined quantitative limitations, while L* denotes brightness on a range from zero to one hundred from black to white. Positive a* corresponds with yellow, negative a* with green, positive b* with blue, and both negative and positive b* with red. One way ANOVA analysis was done which showed significant results between group means (Table 2). Post Hoc Test was performed (Tukey HSD) for multiple comparisons showed statistically significant results with all the groups (table 3). This shows that Neo sealing gel and commercially available gel has less micro- leakage as compared to control group. Neo sealing gel showed better results as compared to commercially available gel.

## Discussion:

Several studies have suggested that the micromotion and abutment screw loosening increases with cyclic loading [7, 8]. Mastication plays a major role in the formation of micro gaps between the implant abutment junctions. Various forces act on the implant abutment junction, such as the shear, tensile and compressive forces, these forces have the ability to loosen the internal screw due to the settling effect over a long period of time [8]. This screw loosening in turn causes micro gaps at the implant abutment junction [9, 10]. As a result, bacteria can readily colonize the free spaces of the implant abutment assembly.

## Conclusion

Based on the findings it can be concluded that using a sealing gel with antibiotics is more effective in controlling bacterial accumulation and micro-leakage as compared to using a sealing gel without antibiotics. The addition of antibiotics to the sealing gel provides an antimicrobial effect that helps to prevent bacterial growth and accumulation, thereby reducing the risk of micro-leakage and subsequent complications. This highlights the importance of using appropriate materials in dental procedures to improve treatment outcomes and reduce the risk of complications.

## Figures and Tables

**Table 1:** Represents the mean L*,a*,b* values for colour analysis from spectrophotometry testing.(group 1-control,group 2- neo sealing gel,group 3- commercially available sealing gel)

**GROUPS**	**PRE**			**POST**			dE*ab
	L*	a*	b*	L*	a*	b*	
1	100.09	0.01	-0.03	100.22	-0.19	-0.17	0.28
2	100.09	0.01	-0.03	100.09	-0.03	-0.04	0.04
3	100.09	0.01	-0.03	100.27	0.01	-0.02	0.17

**Table 2:** Represents one way ANOVA assessment between three different gels.

**COMPARISON**	**df**	**Mean square**	**Significance**
Between groups	2	0.68	0
Within groups	12	0	0
Total	14	-	0
* the mean significant difference is at 0.05 level p=0.00

**Table 3:** Represents tukey post hoc test for multiple comparisons between the 3 groups. (1- Control, 2- Neo sealing gel and 3- Commercially available gel)

**Groups**		**Mean Difference**	**Standard Error**	**Significance**
1	2	0.232	0.0566	0.00*
	3	0.098		
2	1	-0.232	0.0566	0.00*
	3	-0.134		
3	1	0.098	0.0566	0.00*
	2	0.134		
* the mean significant difference is at 0.05 level p=0.00
